# Supplementary material for: Age Related Differences in Monocyte Subsets and Cytokine Pattern during Acute COVID-19—A Prospective Observational Longitudinal Study
Source: Cells. 2021 Nov 30;10(12):3373. doi: 10.3390/cells10123373 (PMC8699549; doi:10.3390/cells10123373)
Supplement: Supplementary file 1 [file cells-10-03373-s001.zip › cells-1419703-supplementary.pdf]

Supplementary Figures:

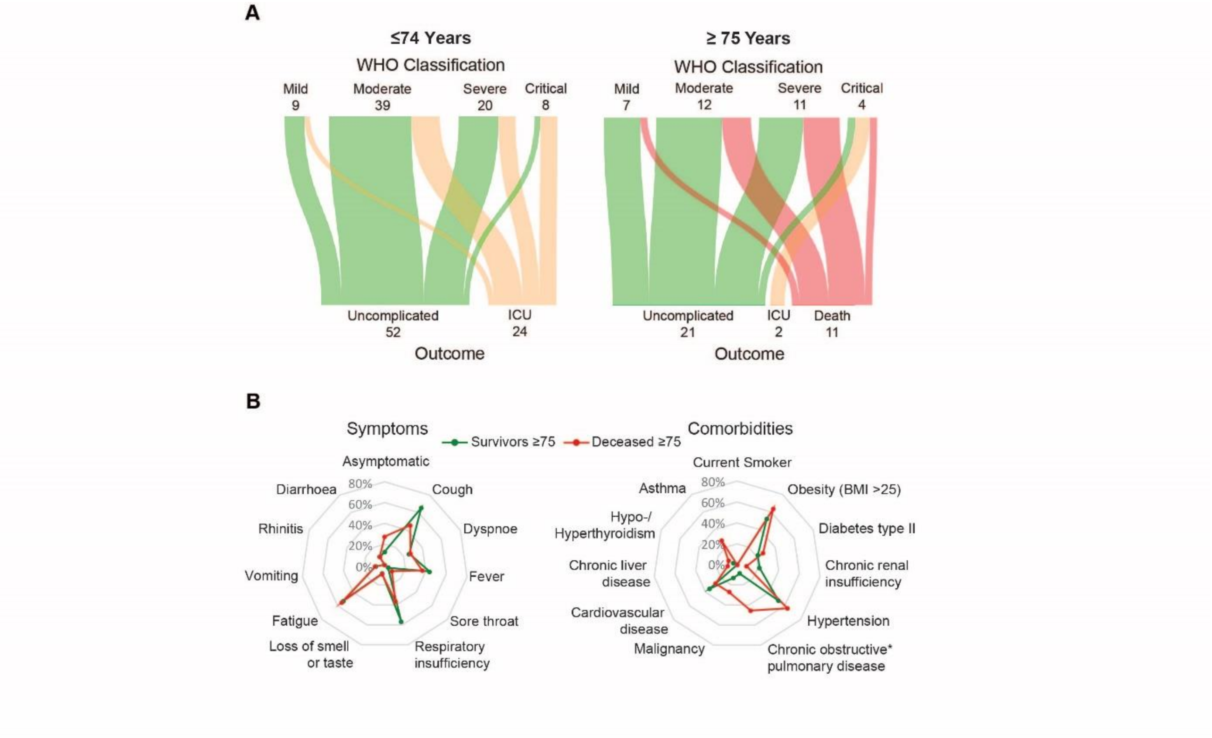

Supplementary Figure S1: Disease severity, outcome, symptoms and comorbidities of young and elderly COVID-19 patients: A) Sankey diagram visualizing WHO disease severity classification at admittance and clinical outcome of COVID-19 patients below (left) and above (right) 75 years of age, B) Percentage of survived and diseased COVID-19 patients above 75 years of age having symptoms (left) or comorbidities (right).

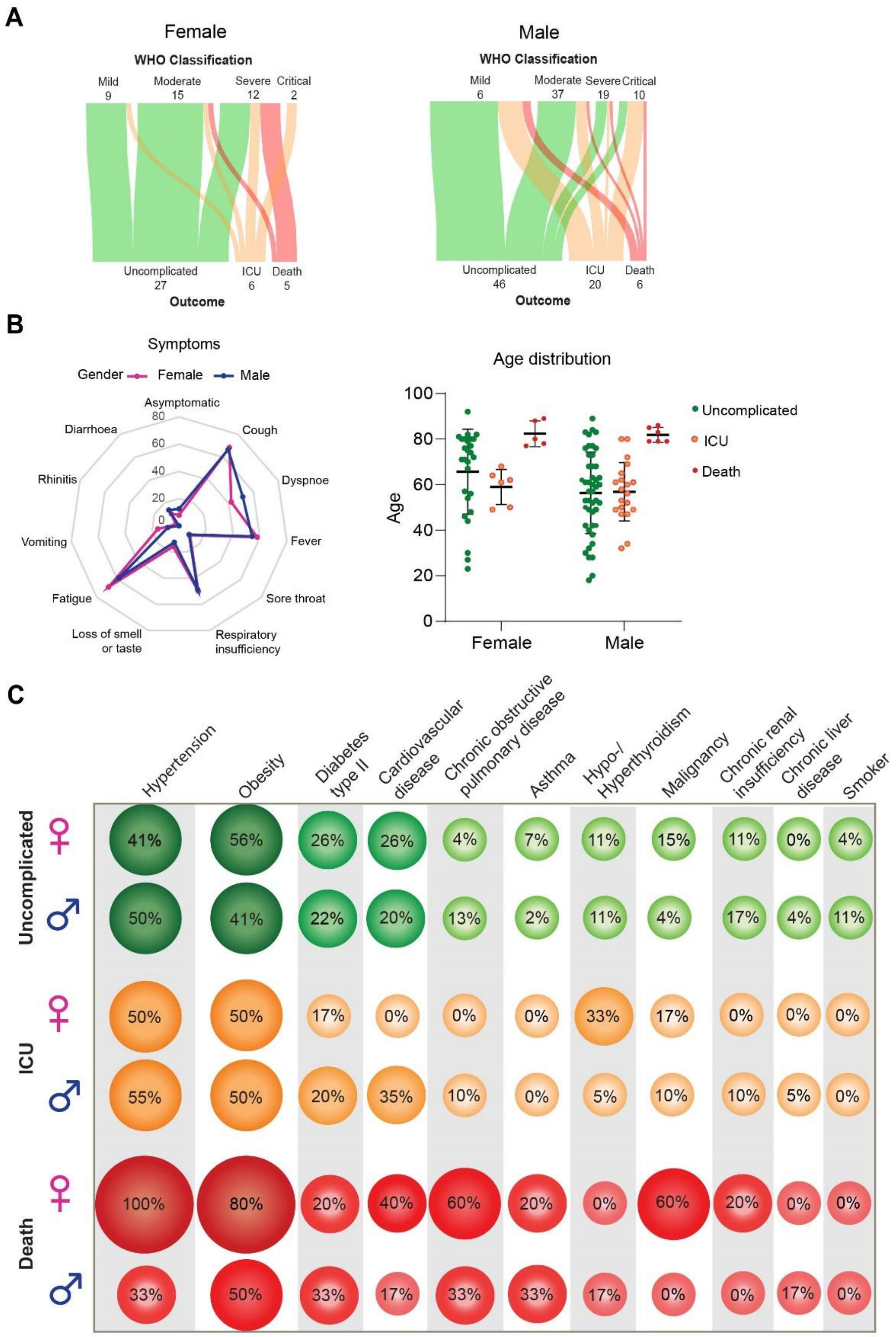

Supplementary Figure S2: Disease severity, outcome, symptoms and comorbidities of female and male COVID-19 patients: A) Sankey diagram visualizing WHO disease severity classification at admittance and clinical outcome of female (left) and male (right) COVID-19 patients, B) Percentage of symptoms experienced by COVID-19 patients (left) and the age distribution in the outcome groups (right) according to sex, C) The prevalence of comorbidities of female and male patients according to the clinical outcome groups.

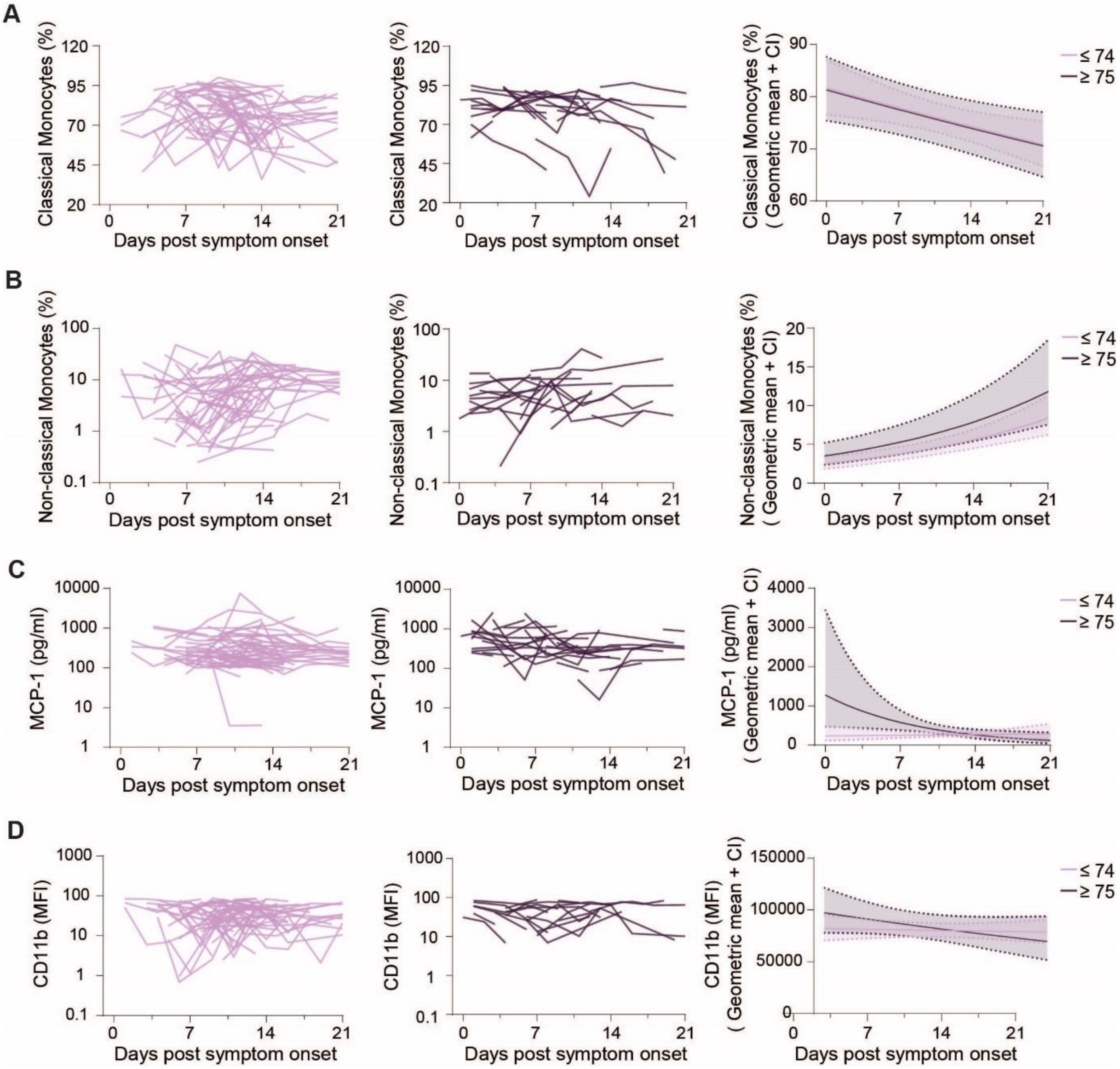

Supplementary Figure S3: Frequencies of monocyte subsets and levels of monocyte activation markers in COVID-19 patients below and above 75 years of age over the disease course: Raw values for individual COVID-19 patients below (left) or above (middle) 75 years of age and modelled values (right) of A) classical monocytes, B) non-classical monocytes, C) MCP-1 and D) activated CD11b.

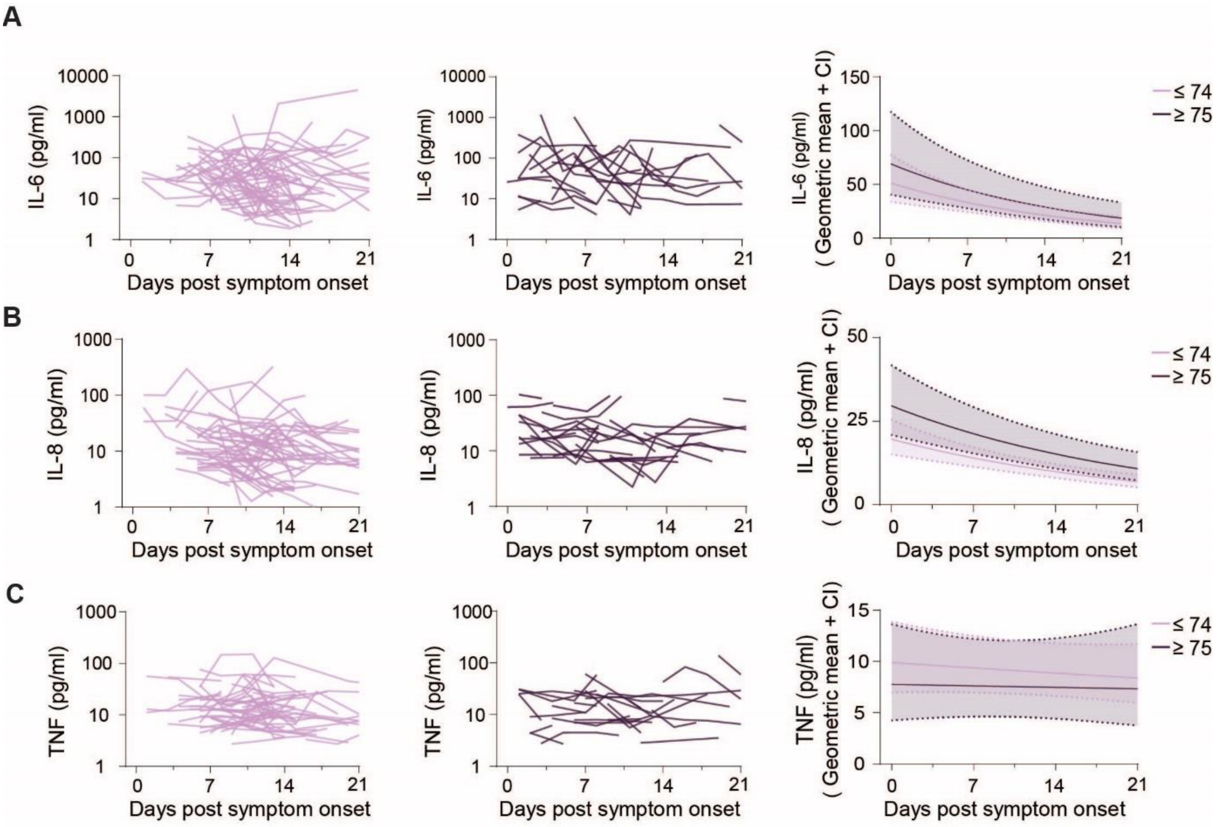

Supplementary Figure S4: IL-6, IL-8 and TNF *levels* of COVID-19 patients below and above 75 years over the disease course: Raw cytokine levels for individual COVID-19 patients below (left) or above (middle) 75 years of age and modelled values (right) of A) IL-6, B) IL-8 and C) TNF *in plasma over the disease course.*

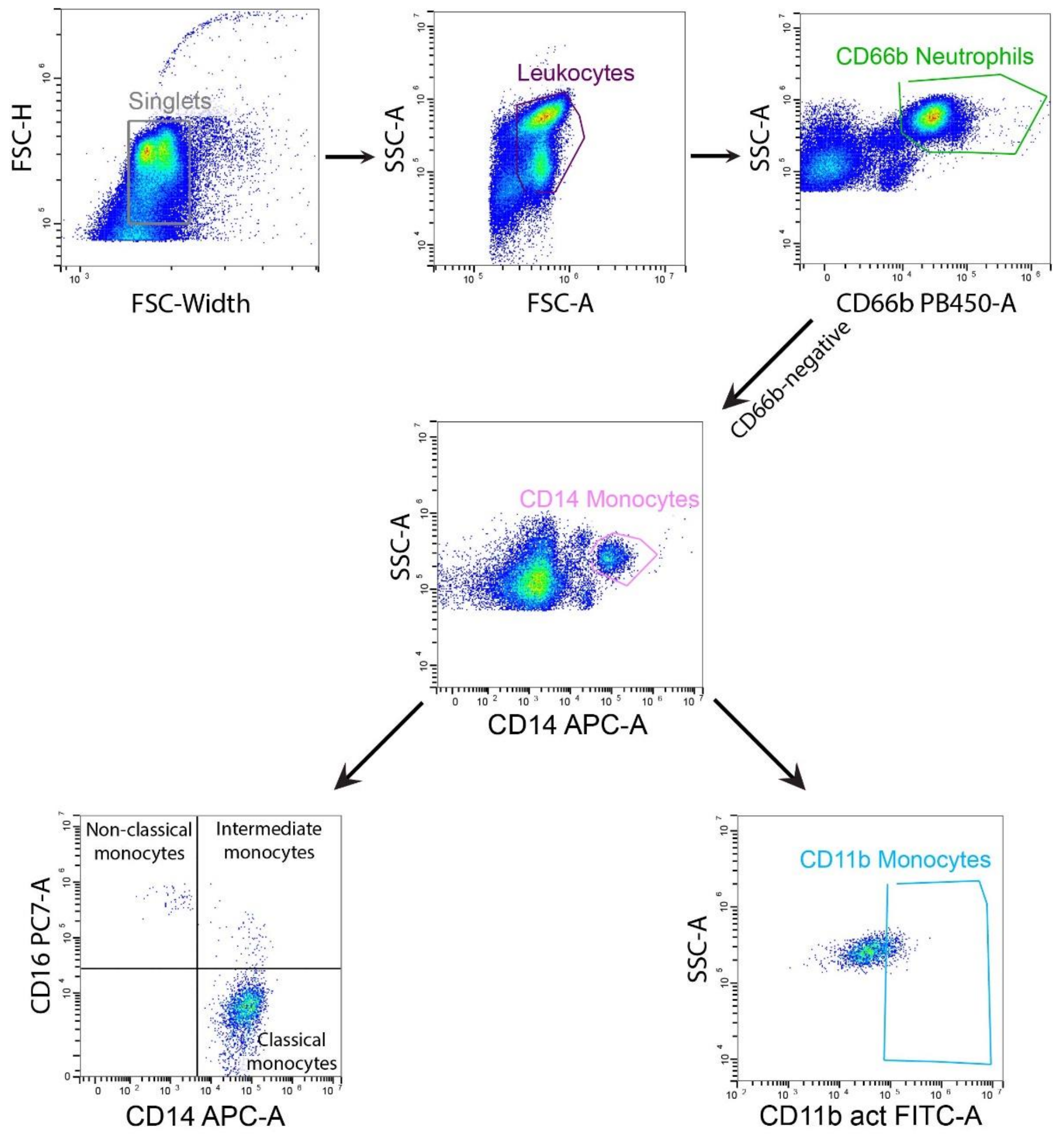

Supplementary Figure S5: Gating strategy of flow cytometry data: Monocytes were defined as being CD66b-negative and CD14-positive. Classical and non-classical monocyte subpopulations were analysed based on the expression of CD14 and CD16. Activated CD11b of monocytes was determined by quantifying the expression of CD11b on CD14-positive monocytes.

Supplementary Table S1: Patient demographics and laboratory parameters at hospital admission

	Missing Data	All (n= 110)	≤ 74 Years (n= 76)	≥ Years (n= 34)	
**Parameter**	**n**	**n (%) Median (IQR)**	**n (%) Median (IQR)**	**n (%) Median (IQR)**	**p-Value***
**Sex**					**p = 0.023**
Male	-	72 (65.5)	55 (72.4)	17 (50.0)	
Female	-	38 (34.5)	21 (27.6)	17 (50.0)	
**Comorbidities**					
Current smoker	32	6 (7.7)	6 (7.9)	0 (0.0)	p = 0.110
Obesity (BMI > 25)	12	54 (55.1)	35 (46.1)	19 (55.9)	p = 0.179
Diabetes type II	-	25 (22.7)	17 (22.4)	8 (23.5)	p = 0.893
Hypertension	1	55 (50.5)	36 (47.4)	19 (55.9)	p = 0.327
Cardiovascular disease (any)	-	26 (23.6)	15 (19.7)	11 (32.4)	p = 0.150
Coronary heart disease	-	14 (12.7)	9 (11.8)	5 (14.7)	p = 0.677
Chronic heart failure	-	8 (7.3)	7 (9.2)	1 (2.9)	p = 0.242
Atrial fibrillation	-	11 (10)	5 (6.6)	6 (17.6)	p = 0.074
Peripheral arterial disease	-	6 (5.5)	3 (3.9)	3 (8.8)	p = 0.298
Chronic obstructive pulmonary disease	-	14 (12.7)	7 (9.2)	7 (20.6)	p = 0.098
Asthma	1	6 (5.5)	3 (3.9)	3 (8.8)	p = 0.306
Hypo- / Hyperthyroidism	1	12 (11.0)	10 (13.2)	2 (5.9)	p = 0.250
Chronic renal insufficiency	-	14 (12.7)	8 (10.5)	6 (17.6)	p = 0.300
Chronic liver disease	-	4 (3.6)	3 (3.9)	1 (2.9)	p = 0.794
Malignancy	-	12 (10.9)	6 (7.9)	6 (17.6)	p = 0.129
**Symptoms**					
Asymptomatic	-	12 (10.9)	6 (7.9)	6 (17.6)	p = 0.129
Cough	-	74 (67.3)	54 (71.1)	20 (58.8)	p = 0.207
Dyspnoea	-	53 (48.2)	44 (57.9)	9 (26.5)	p **= 0.002**
Fever	-	61 (55.5)	47 (61.8)	14 (41.2)	p **= 0.044**
Sore throat	-	11 (10.0)	9 (11.8)	2 (5.9)	p = 0.336
Respiratory insufficiency	-	54 (49.1)	37 (48.7)	17 (50.0)	p = 0.898
Loss of smell or taste	38	15 (20.8)	12 (15.8)	3 (8.8)	p = 0.929
Fatigue	-	68 (61.8)	50 (65.8)	18 (52.9)	p = 0.200
Vomiting	-	12 (10.9)	9 (11.8)	3 (8.8)	p = 0.639
Rhinitis	1	1 (0.9)	1 (1.3)	0 (0.0)	p = 0.508
Diarrhoea	-	14 (12.7)	11 (14.5)	3 (8.8)	p = 0.411
**COVID-19 classification at admission †**					p = 0.622
Asymptomatic / mild	-	15 (13.6)	9 (11.8)	6 (17.6)	
Moderate	-	52 (47.3)	39 (51.3)	13 (38.2)	
Severe	-	31 (28.2)	20 (26.3)	11 (32.4)	
Critical	-	12 (10.9)	8 (10.5)	4 (11.8)	
**Outcome**					**p < 0.001**

Uncomplicated	-	73 (66.4)	52 (68.4)	21 (61.8)	
ICU	-	26 (23.6)	24 (31.6)	2 (5.9)	
Death	-	11 (10.0)	0 (0.0)	11 (32.4)	
**Clinical Characteristics**					
Total hospitalization (days)	1	12 (9.0-21.5)	12 (9.0-18.0)	14 (9.0-29.3)	p = 0.198
Invasive ventilation	-	5 (4.5)	1 (1.3)	4 (11.8)	**p = 0.015**
**Clinical Parameter**					
Hemoglobin (g/dL)	76	13.8 (12.3-14.5)	13.8 (12.4-14.5)	13.4 (12.2-15.0)	p = 105
Red blood cell count ($x10^{12}$/L)	76	4.6 (4.2-4.9)	4.6 (4.2-4.8)	4.5 (3.8-5.2)	**p = 0.001**
Platelet count ($x10^9$/L)	76	190.0 (158.3-252.5)	177.0 (143.3-249.8)	230.5 (179.8-263.3)	p = 0.100
Leukocyte count ($x10^9$/L)	75	5.5 (4.2-8.1)	5.5 (4.1-6.9)	7.2 (5.4-12.0)	p = 0.146
Lymphocyte count ($x10^9$/L)	80	0.8 (0.7-1.3)	0.9 (0.6-1.3)	0.8 (0.7-1.2)	p = 0.113
Neutrophil count ($x10^9$/L)	79	4.4 (3.3-5.3)	4.2 (3.1-5.1)	5.7 (3.8-10.2)	**p = 0.001**
Monocyte count ($x10^9$/L)	80	0.3 (0.2-0.4)	0.3 (0.2-0.3)	0.4 (0.3-0.4)	p = 0.989
Eosinophil count ($x10^9$/L)	80	0.01 (0.0-0.01)	0.01 (0.01-0.01)	0.00 (0.00-0.01)	**p = 0.005**
Basophil count ($x10^9$/L)	80	0.02 (0.01-0.03)	0.02 (0.01-0.03)	0.03 (0.01-0.05)	p = 0.065
C-reactive protein (mg/L)	75	62.5 (38.8-108.8)	57.4 (32.4-99.0)	97.7 (60.4-115.5)	p = 0.870
D-dimer (mg/L)	77	0.6 (0.4-0.9)	0.6 (0.4-0.9)	0.6 (0.5-0.9)	p = 0.758
Prothrombin time (%)	77	101.6 (94.5-109.4)	102.8 (92.6-109.9)	98.0 (95.4-107.6)	p = 0.741
International normalized ratio	77	1.00 (1.00-1.04)	1.00 (1.00-1.05)	1.02 (1.00-1.03)	p = 0.776
Activated partial thromboplastin time (s)	77	35.1 (30.7-37.8)	35.2 (31.0-38.1)	35.1 (29.1-37.1)	p = 0.364

*p<0.05. Nominal variables were compared using the Chi-square test, metric data that were normally distributed were compared using the Student´s T-Test and metric data that were not normally distributed were compared using the Mann Whitney U-Test. The Kolmogorov-Smirnov Test was applied to assess for normality.

† COVID-19 classification according to the guidelines issued by the World Health Organization in mild (fever <38°C, no dyspnoea, no pneumonia), moderate (fever, respiratory symptoms, pneumonia), severe (respiratory distress with respiratory rate ≥30 per minute, oxygen saturation < 93% at rest) and critical (respiratory failure with requirement of mechanical ventilation, requirement of ICU)
BMI: body mass index; ICU: intensive care unit; IQR: interquartile range.

Supplementary Table S2: Antibodies for flow cytometry analyzes

Antibody	Fluorophore	Clone	Final concentration	Company
α-CD66b	Pacific Blue	G10F5	1:75	BioLegend
α-CD14	APC	MØ9	1:60	BD Biosciences
α-CD16	PE-Cyanine7	3G8	1:150	BioLegend
α-CD11b-act	FITC	CBRM1/5	1:60	BioLegend
